# Physiological and Transcriptome Analysis Revealed the Effect of ABA on Promoting Persimmon Fruit Postharvest Deastringency

**DOI:** 10.3390/life15071027

**Published:** 2025-06-27

**Authors:** Han Zhou, Jiao-Jiao Nie, Meng-Lin Ren, Yu-Duan Ding, Ya-Xiu Xu, Qing-Gang Zhu

**Affiliations:** College of Horticulture, Northwest A&F University, Yangling 712100, China

**Keywords:** persimmon fruit, deastringency, ABA treatment, transcriptome analysis

## Abstract

Persimmon (*Diospyros kaki* Thunb.) fruit can accumulate proanthocyanidins (tannins) during development, which causes astringency and affects consumption. The hormone abscisic acid (ABA) has been reported to play a key role in fruit ripening and softening. However, the effect of ABA on postharvest persimmon fruit deastringency remains unclear. In this study, we found that 300 mg/L ABA treatment could decrease the content of soluble tannins, thus leading removal of persimmon fruit astringency. The contents of acetaldehyde and ethanol did not increase during the storage time, indicating that ABA treatment-promoted persimmon fruit deastringency was not due to the acetaldehyde interaction with soluble tannins. Furthermore, the transcriptome analysis showed that 6713 differentially expressed genes (DEGs) were identified, and the WGCNA (weighted gene co-expression network analysis) showed that one module, which comprises 575 DEGs, significantly correlated with the contents of soluble and resoluble tannins. The analysis based on the carbohydrate metabolism pathway indicated that 37 differentially expressed structural genes involved in acetaldehyde metabolism were upregulated by ABA. Real-time quantitative PCR showed that the previously reported key genes, including structural genes and transcription factors, were all upregulated by ABA treatment. The obtained results indicate that ABA treatment, promoting persimmon fruit astringency removal, may occur through gel polymerization of cell wall materials with soluble tannins.

## 1. Introduction

Persimmon (*Diospyros kaki* Thunb.) is a kind of fruit that originates from China and is currently cultivated worldwide [1]. The most notable feature of persimmon fruit is that it accumulates proanthocyanidins (PAs, also known as condensed tannins, CTs), of which soluble tannins cause fruit astringency. In general, in the fruit industry, persimmon fruit is simply classified into astringent type and sweet type based on whether they have a bitter taste after ripening [1]. Most of the cultivated cultivars are of the astringent type, so technologies for astringency removal need to be developed for persimmon fruit consumption.

Several artificial postharvest treatments have been developed to remove astringency, including highly concentrated CO_2_ treatment [2], ethylene treatment [3], hot water treatment [4], and ethanol treatment [5]. Three main theories have been proposed to explain the mechanisms of fruit deastringency, including condensation theory, gelation theory, and dilution theory [6]. The condensation theory posits that acetaldehyde can combine with tannins to convert soluble tannins into insoluble tannins, which causes astringency removal. The anaerobic condition plays an important role in this process [6]. Transcriptome analysis on various cultivars indicated that the differences in acetaldehyde metabolism involved in different persimmon cultivars astringency removal efficiency treated by CO_2_ [7]. Cell wall materials could react with soluble tannins by gel polymerization to make persimmon fruit deastringency, which was considered a gelation theory of fruit deastringency [4]. While the dilution theory explains the density of tannin cells, the fruit flesh decides whether the persimmon fruit is astringent or non-astringent type [6]. Although there are many reported perspectives for understanding persimmon fruit astringency removal, further exploration is required.

Abscisic acid (ABA) is an important phytohormone involved in fruit ripening and softening [8,9]. Many studies have reported that ABA plays an important role in fruit ripening and softening through regulating the coloration, aroma release, texture, and flavor substances changes [9,10,11,12,13]. In our previous study, we found that ethylene treatment could promote the metabolism of soluble tannins, facilitating the removal of astringency in persimmon [3,14]. The effectiveness of ABA treatment on persimmon fruit deastringency has not yet been reported.

In our previous studies, we found that ABA treatment could promote persimmon fruit softening [9,15]. At the same time, we also found that ABA could decrease the content of soluble tannins, promoting persimmon fruit astringency removal. So, physiological and transcriptome analysis were employed in order to explore the potential mechanism of ABA involvement in tannin metabolism.

## 2. Materials and Methods

### 2.1. Plant Material and Treatments

Persimmon fruit (*Diospyros kaki* cv. Fupingjianshi) was harvested at the mature stage with over 80% surface yellow coloration, in 2022, from an orchard in Fuping, Shaanxi, China. A total of 100 persimmon trees with the same growing conditions that are adjacent to each other (10 rows with 10 trees in each row) were selected for fruit harvesting. A total of 600 fruit with the same degree of maturity were randomly picked from the 100 trees, of which 400 uniform fruit without visual defects were selected and randomly divided into two groups for the following treatments: (I) sprayed with 300 mg/L ABA (the concentration of ABA was in accordance with our previous reports, [9,15]) four times (0 h, 6 h, 12 h, and 18 h); (II) sprayed with water as the control (CK). All fruit was placed onto two separate tables that did not affect each other during processing. After treatment, the fruit was stored at 20 °C with 60% relative humidity and collected at 0, 1, 2, 4, 6, 10, 14, and 20 days, respectively. At each sampling time, twelve fruits (three biological replicates, with four fruits for each biological replicate) from each treatment were cut and frozen in liquid nitrogen and stored at −80 °C for future use.

### 2.2. Soluble and Resoluble Tannin Contents

Soluble and resoluble tannins content were measured using the Folin–Ciocalteu reagent according to the methods described by Yin et al. [3], using the frozen flesh with three biological replicates. The results were calculated using a standard curve of tannic acid equivalents g^−1^ fresh weight.

### 2.3. Ethanol and Acetaldehyde Measurements

The contents of fruit endogenous ethanol and acetaldehyde were determined using an Agilent 6890N gas chromatograph (Agilent, Santa Clara, CA, USA) according to the method described by Min et al. [2]. A total of 2 g of fruit powder was extracted and homogenized with 5 mL of saturated NaCl, of which 3 mL of the mixture was drawn into a headspace extraction bottle (20 μL of sec-butanol was added in each sample as an internal reference), and then placed in a 60 °C water bath for 1 h. A total of 1 mL of headspace gas was injected into the gas chromatograph after heating. The temperature of the sample inlet, oven, and detector of the gas chromatograph were set at 150 °C, 100 °C, and 160 °C, respectively. The results were determined by the standard curve of ethanol and acetaldehyde. Each measurement was performed with three biological replicates.

### 2.4. RNA Isolation and Transcriptomic Analysis

Total RNA was extracted from fruit flesh using the CTAB (hexadecyl trimethyl ammonium bromide) protocol developed by Chang et al. [16]. The RNA samples from two treatments (0 d, 1 d, 2 d, 4 d, 6 d, 10 d, ABA treatment named as A1~A10, and the control named as CH1~CH10) were sent for RNA-seq analysis by Biomarker Technologies Corporation (Beijing, China). The RNA purity and concentration were measured by the NanoDrop 2000 (Thermo Fisher Scientific, Wilmington, DE, USA). The NEBNext Ultra^TM^ RNA Library Prep Kit for Illumina (NEB, Ipswich, MA, USA) was applied to construct the RNA-seq libraries, and the Illumina NovaSeq 6000 (Illumina, San Diego, CA, USA) was used for sequencing. The clean, high-quality data was analyzed after sequencing. HISAT2 [17] was applied to map the clean reads to the persimmon genome [1]. Quantification of the gene expression levels was estimated by fragments per kilobase of transcript per million (FPKM) fragments mapped. The FPKM is not zero named the expressed genes. DESeq2 [18] was performed to analyze the differentially expressed genes of the two groups. Genes with *p*-value < 0.05 and |log2(Fold change) |> 1 were considered as differentially expressed genes (DEGs) and were annotated with GO terms.

### 2.5. Weighted Gene Co-Expression Network Analysis (WGCNA)

Weighted gene co-expression network analysis was performed using the R (4.3.1) package WGCNA [19] combined the DEGs (FPKM of average three replicates > 1) and the contents of soluble tannins and resoluble tannins. The co-expression networks were visualized with Cytoscape (v.3.9.1) [20]. The DEGs in the key module were further analyzed by Kyoto Encyclopedia of Genes and Genomes (KEGG, https://www.genome.jp/kegg) and Gene Ontology (GO) functional calsfifications.

### 2.6. cDNA Synthesis and RT-qPCR Analysis

The genomic DNA removal and cDNA synthesis were accomplished using the Prime Script RT reagent Kit with gDNA eraser (TaKaRa, Dalian, China) according to the manufacturer’s protocol. SYBR PrimeScript RTPCR kit II (TaKaRa, Dalian, China) was applied for RT-qPCR with an iCycler iQ5 (Bio-Rad, Hercules, CA, USA), and the method described by Zhu et al. [1] was employed. RT-qPCR specific primers of involved genes (*DkADH1*, *DkPDC2*, *DkPK1*, *DkERF9/10/18/19*, and *DkWRKY1*) were designed by Primer3 input (v. 0.4.0) and are listed in Table S1.

### 2.7. Statistical Analysis

The statistical significance of differences was determined by an ANOVA test and least significant difference (LSD) for multiple comparisons by DPS 2.05 (Zhejiang University). Figures were drawn using Origin 2024 (Microcal software) and Adobe Photoshop CC 2023.

## 3. Results

### 3.1. Effect of ABA Treatment on Persimmon Fruit Postharvest De-Astringency

**Figure 1:**
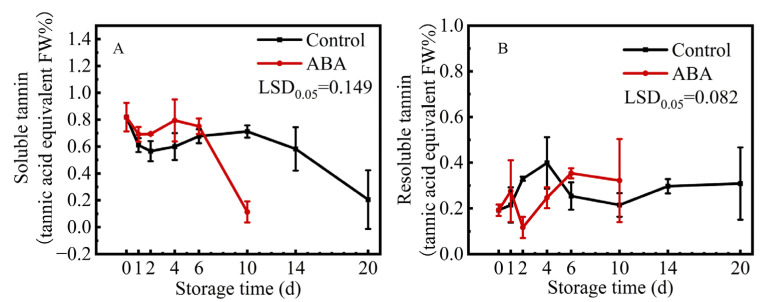
Changes in contents of soluble tannins (**A**) and resoluble tannins (**B**) in ‘Fupingjianshi’ persimmon fruit in response to ABA treatment. Error bars ± SE from three replicates. LSDs represent the least significant difference at *p* = 0.05.

Mature ‘Fupingjianshi’ persimmon fruit were astringent at harvest, and the soluble tannin content was approximately 0.82%. ABA treatment caused a decrease in soluble tannins by 0.11% after 10 d, compared to the content of soluble tannins in the control fruit, which was 0.71% at 10 d and 0.23% at 20 d (Figure 1A). While the content of resoluble tannins (insoluble in water and soluble in hydrochloric acid could then be measured) showed an opposite trend of changing, increased at the beginning and then maintained at a stable level (Figure 1B). These results indicated that ABA treatment could decrease the content of soluble tannins, leading to persimmon fruit astringency removal.

### 3.2. Effects of ABA Treatment on Endogenous Ethanol and Acetaldehyde Contents

**Figure 2:**
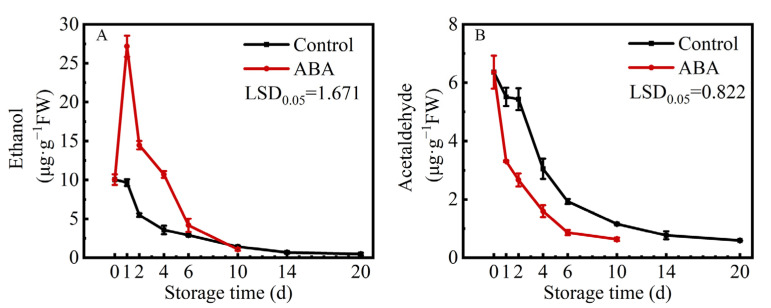
Changes in contents of ethanol (**A**) and acetaldehyde (**B**) in ‘Fupingjianshi’ persimmon fruit in response to ABA treatment. Error bars ± SE from three replicates. LSDs represent the least significant difference at *p* = 0.05.

Compared to the control, the content of ethanol in ABA-treated fruit showed a rapid increase from 10.03 μg/g on 0 d to 27.18 μg/g on 1 d. Then, the content of ethanol exhibited a downward trend during storage in both treatments (Figure 2A). Additionally, the content of acetaldehyde decreased quickly from the beginning in the control and ABA treatment (Figure 2B). These two results showed that ABA accelerated persimmon fruit astringency removal not by inducing acetaldehyde production.

### 3.3. RNA-Seq and Weighted Gene Co-Expression Network Analysis

**Figure 3:**
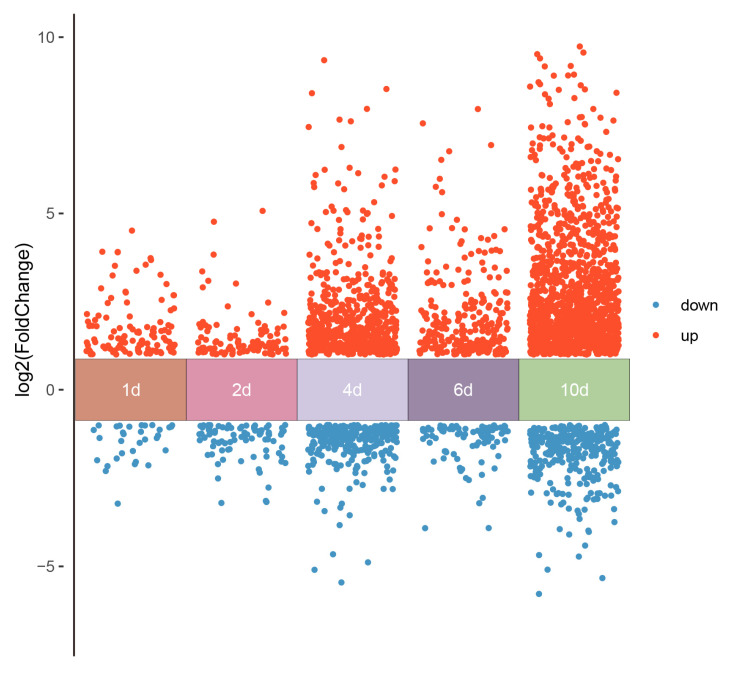
Summary of DEGs of RNA-seq analysis of persimmon fruit treated by Control and ABA. The DEGs were 145, 188, 877, 344, and 1590 on 1 d, 2 d, 4 d, 6 d, and 10 d, respectively.

In order to understand how the mechanism of ABA regulates tannin metabolism, transcriptomic sequencing was performed to explore the DEGs. For transcriptome analysis, every sample obtained an average of about 6.0 Gb of clean data, with ≥97% (Q20) and ≥92% (Q30), respectively (Table S2). In total, 32,505 expressed genes were identified, of which 6713 were DEGs (a threshold of the log2 |Fold Change| ≥ 1 and *p*-adj < 0.05) between the control and ABA treatment (Figure 3).

**Figure 4:**
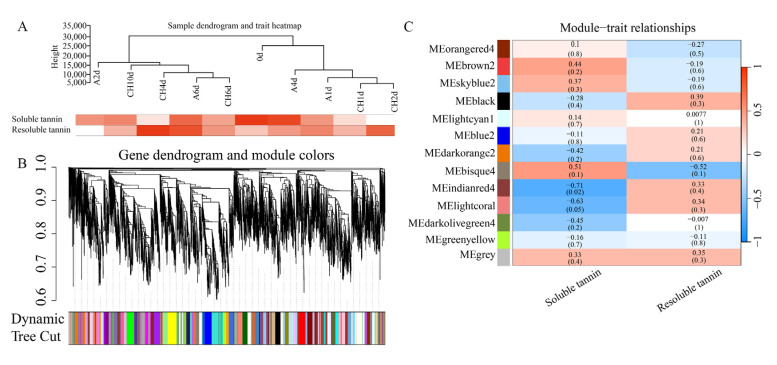
Sample cluster analysis and the construction of the co-expression modules by WGCNA based on physiological data and RNA-seq data. (**A**) Sample dendrogram and trait heatmap based on physiological data and gene expression data. (**B**) Clustering dendrograms of genes, with dissimilarity based on the topological overlap, together with assigned module colors. (**C**) Module−sample association, module−sample correlations, and corresponding *p* values.

To further identify the related genes involved in the metabolism of tannin, WGCNA was conducted using 26,858 genes with FPKM >1 of all sequenced points and the contents of soluble and resoluble tannins. To elucidate the relationship between the corresponding gene expression data and the content of tannin, the sample dendrogram and trait heatmap were visualized (Figure 4A). The genes with the same expression pattern are grouped within the same cluster dendrogram (Figure 4B). Thirteen modules were obtained, of which the Indianred module (containing 575 DEGs) showed significant correlations with soluble and resoluble tannins, with Pearson correlation coefficients of −0.71 and 0.33 (Figure 4C), respectively.

**Figure 5:**
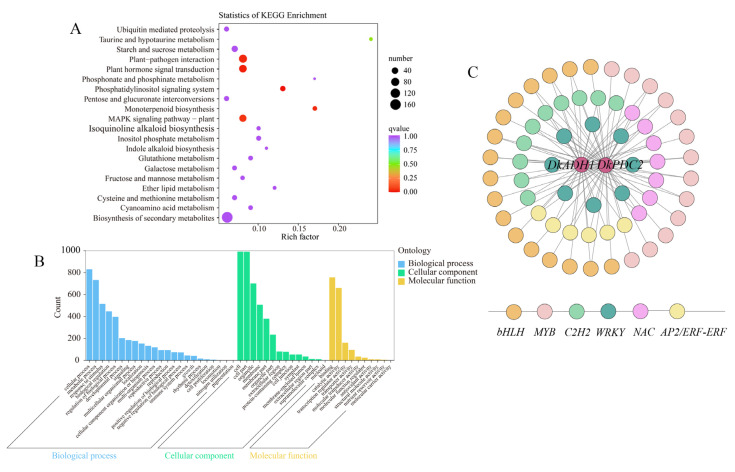
The GO, KEGG pathway enrichment and network analysis of DEGs from the Indianred module. (**A**) KEGG pathway analysis of DEGs. (**B**) Go classification of DEGs. (**C**) Co-expression network analysis of *DkADH1* and *DkPDC2*. The two structural genes were presented in circles in red. Different families of TFs were presented by circles with different colors.

The functional annotation of the DEGs in the Indianred module was investigated using KEGG analysis. Through pathway enrichment, the DEGs were mainly involved in the biosynthesis of secondary metabolites, plant–pathogen interaction, and plant hormone signal transduction (Figure 5A). GO functional classification of the DEGs in the Indianred module showed that the significant enrichment terms for biological processes were mainly identified as cellular process, metabolic process, and responses to stimulus and others. In the cellular component, the cell, cell parts, and organelles were mainly identified. In the molecular category, binding, catalytic activity, and transcription regulator activity were the main terms (Figure 5B). In previous studies, *DkADH1* and *DkPDC2* were identified as key structural genes involved in persimmon fruit deastringency [1,2,21,22], the regulatory network was constructed between these two genes and co-expressed (|Cor| < 0.9 and *p* < 0.05) transcription factors (TFs) (Figure 5C). A total of 61 TFs, including bHLH, MYB, C2H2, WRKY, NAC, and AP2/ERF families (Table S3).

### 3.4. Differential Responses of Carbohydrate Metabolism Related Genes to ABA Treatment in Persimmon Fruit

**Figure 6:**
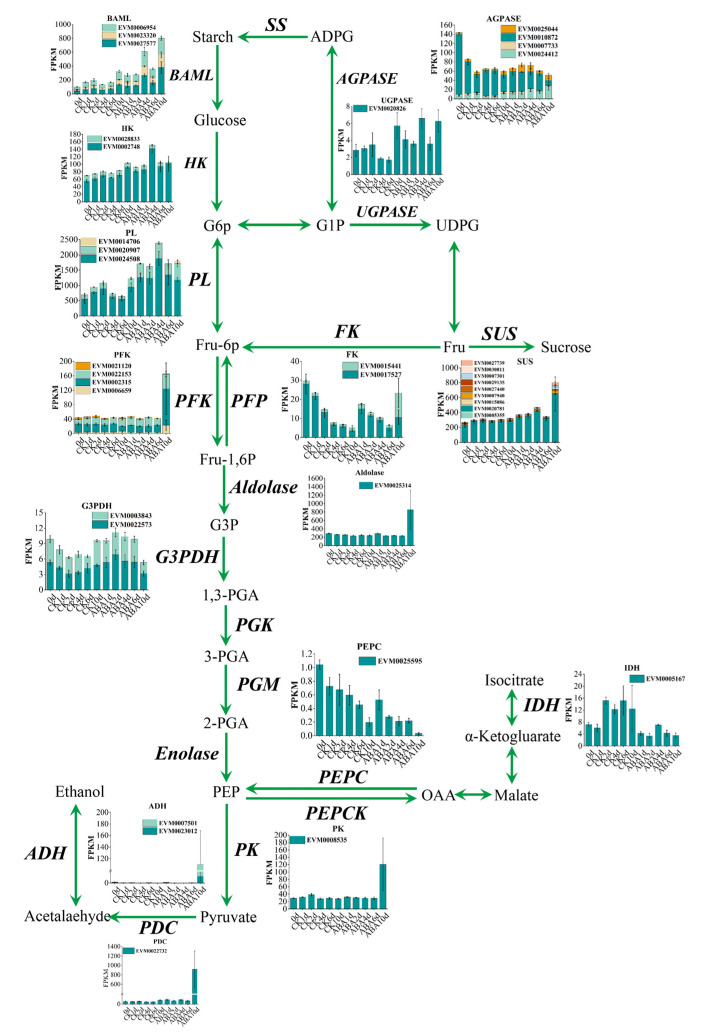
Carbohydrate metabolism profile in ‘Fupingjianshi’ persimmon fruit treated by ABA and control. The expression profiles of DEGs represent the FPKM of DEGs.

Previous studies have found that carbohydrate metabolism plays an important role in the persimmon fruit deastringency [7]. As shown in Figure 6, 37 genes related to the carbohydrate metabolism were identified, most of which were upregulated in ABA treatment fruit, especially the PFK (EVM0002315, EVM0006659, and EVM0022153), PFP (EVM0015441), and Aldolase (EVM0025314) were highly expressed in ABA group, indicating they may play important roles in carbohydrate metabolism treated by ABA. Three genes, *DkADH1* (EVM0007501), *DkPDC2* (EVM0022732), and *DkPK1* (EVM0008535), which were proven to be the key structural genes involved in persimmon deastringency [22,23], showed higher expression levels in the ABA treatment fruit than in the control group.

### 3.5. RT-qPCR Validation of RNA-Seq Data

**Figure 7:**
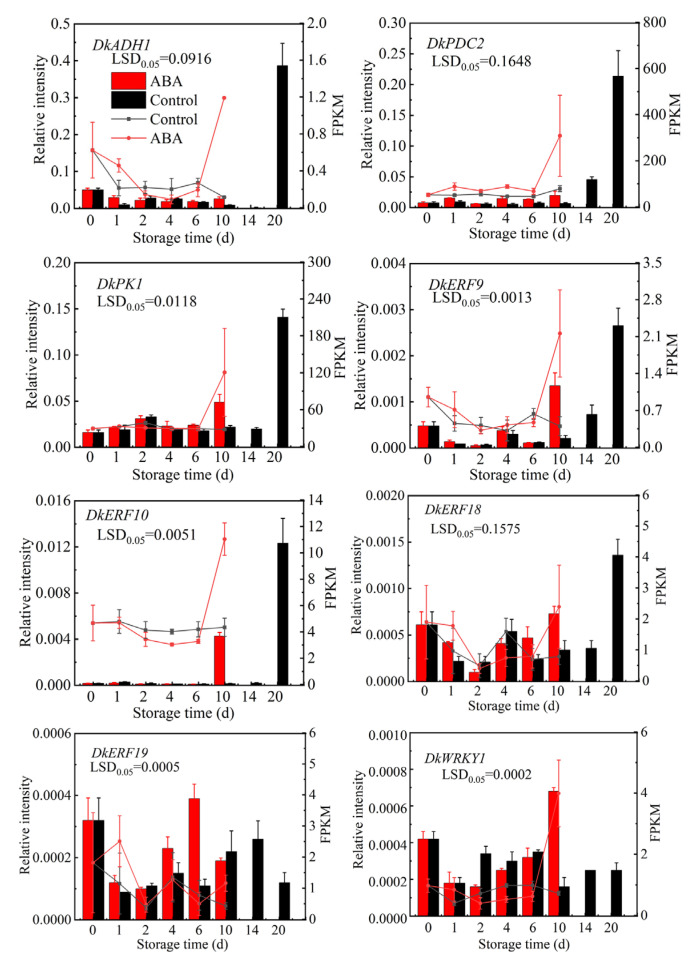
Quantitative RT-qPCR validations. Eight genes were previously reported to be key structural genes and transcription factors involved in persimmon fruit deastringency. The line chart illustrates the FPKM values obtained from the transcriptome data, and the histogram represents the RT-qPCR data. Values are means (±SE) from three biological replicates.

The eight DEGs (including *DkERF9*, *DkERF10*, *DkERF18*, *DkERF19*, *DkWRKY1*, *DkADH1*, *DkPDC2*, and *DkPK1*), which are known to play important roles in persimmon fruit deastringency [1,22], were selected for expression level analysis by RT-qPCR to validate the accuracy of the RNA-seq data (Figure 7). All of these genes were upregulated by ABA treatment, which is consistent with the expression patterns analyzed by RNA-seq.

## 4. Discussion

Persimmon fruit is unique due to its accumulation of condensed tannins, and soluble tannins cause fruit astringency, which affects consumers’ taste and reduces consumer appeal [1]. Therefore, different technologies have been developed to reduce the content of soluble tannins in persimmon fruit to make it easy to eat, including highly concentrated N_2_ and CO_2_ treatment [2,5,21,22], dipping in hot water [24], and ethylene treatment [3], which all can result in persimmon fruit deastringency. ABA, as a plant hormone, plays an important role in different plant development processes, abiotic stress responses, fruit ripening, leaf abscission, and others [11,15,25]. In this study, ABA treatment was found to promote persimmon fruit deastringency, which can decrease the content of soluble tannins during persimmon fruit storage, and expands our knowledge about the function of ABA. Three theories were proposed by different researchers about the persimmon fruit astringency removal, of which gelation theory indicated that cell wall materials react with tannins to make the persimmon fruit astringency removal [4,6]. In our previous studies, the content of ethanol and acetaldehyde in persimmon fruit showed a rapid increase when treated with high concentrations of CO_2_ or ethylene, which rapidly accelerated persimmon fruit deastringency [2,3,26]. However, in this study, the content of acetaldehyde in ABA-treated fruit decreased after treatment, and we found that ABA treatment could promote persimmon fruit softening quickly [9,15]. This suggests that ABA may play a role in reducing astringency in persimmon fruits. This reduction can be attributed to the gel polymerization of cell wall materials that react with tannins, rather than the combination of acetaldehyde with soluble tannins.

In this study, like ethylene [3], the ABA treatment accelerated the decrease in soluble tannins, leading to persimmon fruit astringency removal, which suggests that the metabolism of tannins may be regulated by many plant hormones. The number of DEGs increased gradually during fruit storage based on the RNA-seq analysis. KEGG and GO analysis showed that the DEGs related to fruit deastringency were mainly enriched in metabolic processes, indicating that the secondary metabolites may play key roles in tannin metabolism. Some TFs, belonging to different families, had been identified to be involved in regulating persimmon fruit astringency removal by regulating the metabolism of acetaldehyde [1,2,23,26]. In this study, 61 TFs from different families were expected to target *DkADH1* and *DkPDC2*, involved in persimmon fruit deastringency. Except for ERF, MYB, and WRKY, other TF families like bHLH, C2H2, and NAC were significantly correlated with the expression of *DkADH1* and *DkPDC2*, which provides new insights into the mechanism of persimmon fruit astringency removal.

*DkERF9/10/18/19* and *DkWRKY1* are key transcription factors involved in the persimmon fruit deastrigency response to high-CO_2_ treatment, targeting *DkADH1* and *DkPDC2*, respectively [2,22,23]. While the RNA-seq and RT-qPCR showed that they all responded to ABA treatment, indicating, except for the fruit deastrigency, that they may be involved in other biological pathways, which need further exploration.

In conclusion, ABA treatment could decrease the content of soluble tannins and promote persimmon fruit astringency removal. Transcriptomic analysis showed that the genes involved in carbohydrate metabolism were upregulated by ABA. However, the contents of ethanol and acetaldehyde did not increase after ABA treatment, indicating that ABA treatment-promoted persimmon fruit astringency removal may be through gel polymerization of cell wall materials reacting with tannins. The previously reported genes involved in persimmon fruit deastringency also responded to ABA treatment, indicating that they may participate in other biological pathways.

## Data Availability

Data are available upon request.

## Supplementary Materials

The following supporting information can be downloaded at: https://www.mdpi.com/article/10.3390/life15071027/s1, Table S1. Primer sequences used for RT-qPCR; Table S2. All samples were evaluated for sequencing data quality. Table S3. Transcription factors with a pearson correlation coefficient higher than 0.9 for *DkADH1* and *DkPDC2*.
